# Eosinophils are key regulators of perivascular adipose tissue and vascular functionality

**DOI:** 10.1038/srep44571

**Published:** 2017-03-17

**Authors:** Sarah B. Withers, Ruth Forman, Selene Meza-Perez, Daniel Sorobetea, Kasia Sitnik, Thomas Hopwood, Catherine B. Lawrence, William W. Agace, Kathryn J. Else, Anthony M. Heagerty, Marcus Svensson-Frej, Sheena M. Cruickshank

**Affiliations:** 1 Institute of Cardiovascular Sciences, University of Manchester, Manchester, United Kingdom; 2 Faculty of Biology, Medicine and Health, University of Manchester, Manchester, United Kingdom; 3 Immunology section, Lund University, BMC D14, SE-22184 Lund, Sweden; 4 University of Alabama at Birmingham, Birmingham, Alabama, USA; 5 Section for Immunology and Vaccinology, Danish Technical University, Veterinary Institute, Bülowsvej 27, DK-1870, Copenhagen, Denmark

## Abstract

Obesity impairs the relaxant capacity of adipose tissue surrounding the vasculature (PVAT) and has been implicated in resultant obesity-related hypertension and impaired glucose intolerance. Resident immune cells are thought to regulate adipocyte activity. We investigated the role of eosinophils in mediating normal PVAT function. Healthy PVAT elicits an anti-contractile effect, which was lost in mice deficient in eosinophils, mimicking the obese phenotype, and was restored upon eosinophil reconstitution. *Ex vivo* studies demonstrated that the loss of PVAT function was due to reduced bioavailability of adiponectin and adipocyte-derived nitric oxide, which was restored after eosinophil reconstitution. Mechanistic studies demonstrated that adiponectin and nitric oxide are released after activation of adipocyte-expressed β3 adrenoceptors by catecholamines, and identified eosinophils as a novel source of these mediators. We conclude that adipose tissue eosinophils play a key role in the regulation of normal PVAT anti-contractile function.

Most of the peripheral circulation is invested by a layer of perivascular adipose tissue (PVAT)^1^, which comprises adipocytes, stromal cells and immune cells. In health, PVAT confers an anti-contractile effect on the vasculature through a balance of adipocyte-derived pro- and anti-contractile factors (including adiponectin^2^) and immune cell populations^3^, as well as contributing to the regulation of physiological processes, including glucose homeostasis and lipid metabolism. In response to sustained caloric excess, there is adipocyte enlargement, hypoxia and subsequent PVAT inflammation leading to increased arterial tone^4^, which has profound effects on peripheral resistance^5^ and nutritive flow^6^, thereby linking obesity-associated hypertension^7^ and type 2 diabetes with vascular dysfunction^8^.

The involvement of immune cells in metabolic events in adipose tissue has come to the forefront of obesity research (reviewed in ref. 9). The contribution of eosinophils to the regulation of physiological events in these tissues, under steady state and in the inflammatory setting, is undefined; increased knowledge in this area is an unmet need with important implications for the treatment of obesity-associated disorders. Historically, eosinophils have been viewed as end-stage effector cells associated with Th2 inflammatory disorders such as parasitic infections and allergies, where they become activated and release cytotoxic granule proteins^10^. However, recent reports demonstrate that eosinophils are steady state constituents of the cellular pool in several organs, including the gastrointestinal tract^11^ and adipose tissue, and play a role in metabolic homeostasis^12^. Despite this, little attention has been paid to the direct role that eosinophils may play in adipose tissue function. We employed mouse models of eosinophil-deficiency and reconstitution, complemented by *in vitro* studies, to address the significance of eosinophils on PVAT function and vascular reactivity. For the first time, we have identified a central role for eosinophils in the maintenance of healthy PVAT functionality. Mechanistically, we define the release of nitric oxide as well as adiponectin, as central in regulation of PVAT anti-contractile function, and importantly identify the eosinophil as a key cell type controlling the release of these mediators via catecholamine mediated-activation of adipocyte-expressed β3 adrenoceptors.

## Results

### Healthy PVAT exerts an anti-contractile effect that is lost in obesity

**Figure 1:**
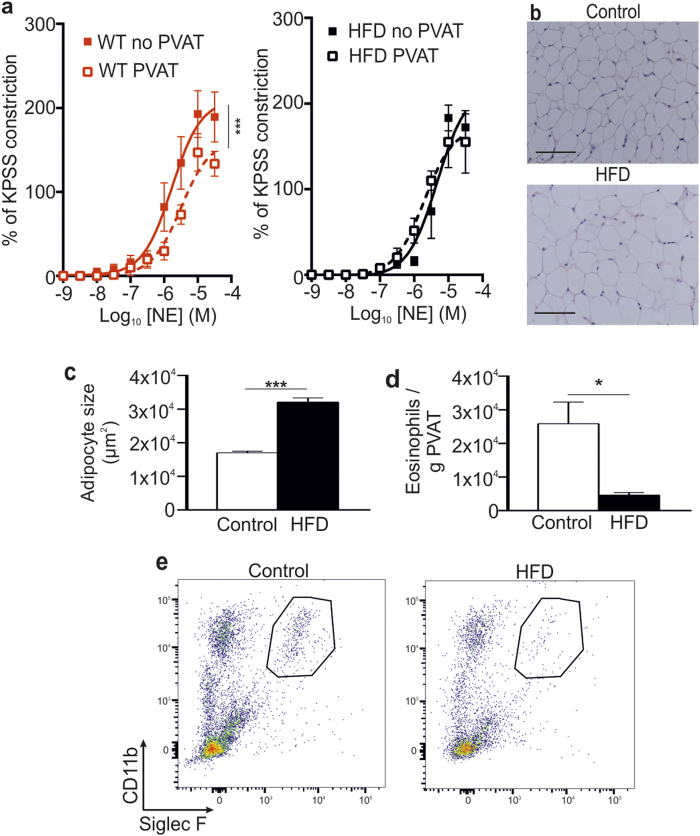
Obese mice have impaired vascular reactivity. Age-matched WT and HFD WT mice were analyzed for vascular reactivity and adipose tissue alterations. **(a)** The anti-contractile effect of PVAT observed in WT mice is lost in HFD mice (mean ± SEM from one experiment; n = 8 (WT) and 5 (HFD); P = NS, two-way ANOVA) (no PVAT (-■-) and PVAT (-□-)). **(b)** Representative H&E staining of mesenteric adipose tissue from control and HFD mice, and **(c)** analysis of adipocyte size (HFD: n = 5, and WT: n = 8; ***P < 0.0001, Student’s t-test). Scale bar shows 100 μm. **(d)** Mesenteric adipose tissue of HFD and control mice was analyzed for number of eosinophils (mean ± SEM; HFD: n = 5, and WT: n = 8; *P = 0.0113, Student’s t-test). (**e**) Representative flow cytometric plots of mesenteric adipose tissue eosinophils from control and HFD mice.

Initially, we compared the vascular reactivity in healthy twelve-week old C57BL/6 mice fed standard chow to mice on a high fat diet (HFD). In mice on standard chow, contractile responses of small mesenteric arteries (approximately 200 μm internal diameter) to cumulative doses of norepinephrine (NE) showed that vascular constriction was reduced in the presence of PVAT, compared with vessels from the same mouse in the absence of PVAT (P = 0.001; Fig. 1a). In contrast, in age-matched obese C57BL/6 mice fed a HFD, the anti-contractile capacity of PVAT was completely abolished, with no difference in contractility to NE whether PVAT was intact or removed (Fig. 1a).

Histological analyses of PVAT demonstrated significant adipocyte hypertrophy in HFD mice compared with standard chow fed control mice (P < 0.0001; Fig. 1b,c). Furthermore, immunohistochemical and flow cytometric analyses of enzymatically digested mesenteric adipose tissue demonstrated a significant reduction in the number of eosinophils present in HFD mice compared with chow fed age-matched controls (P = 0.0113; Fig. 1d,e and data not shown), consistent with previous reports^12^. Thus, impaired vascular function in HFD mice is associated with a dramatic reduction in the number of adipose tissue eosinophils.

### Eosinophils contribute directly to the regulation of vascular tone

#### Anti-contractile PVAT function is lost in eosinophil-deficient ΔdblGATA-1 mice

**Figure 2:**
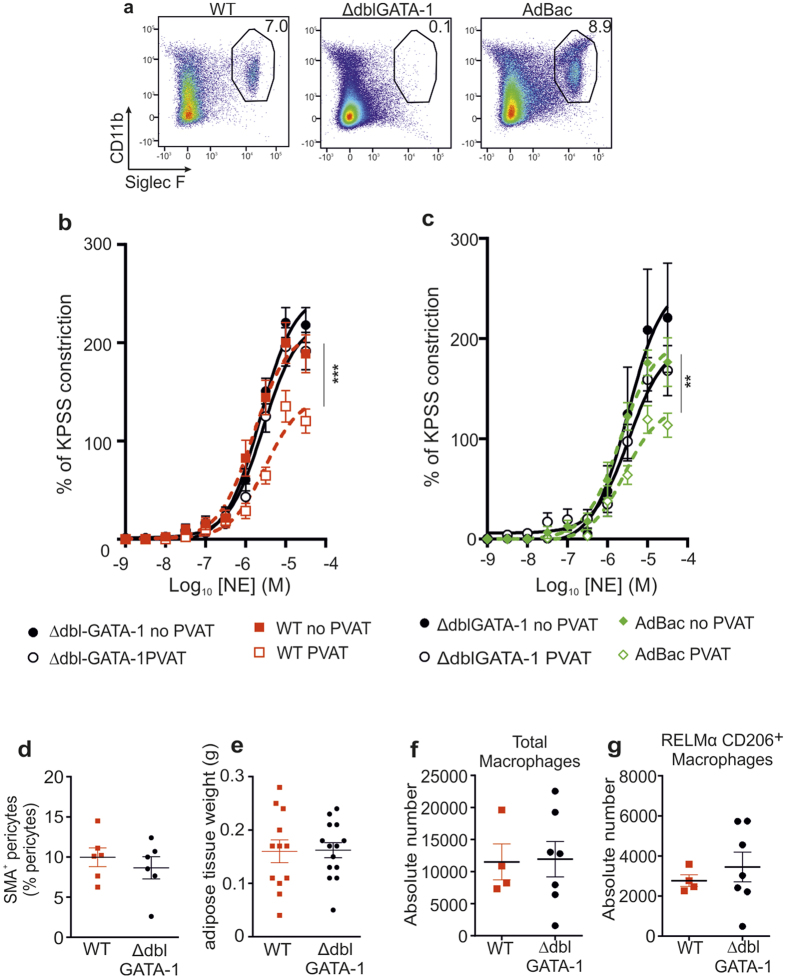
Eosinophil-deficient mice have impaired vascular reactivity which is restored by *in vivo* reconstitution with eosinophils but has no effect on other immune cell populations. (**a**) Flow cytometric analysis of mesenteric adipose tissue eosinophils from WT, ∆dblGATA-1 and eosinophil-reconstituted (AdBac) mice (n = 11, 6 and 5, respectively). (**b**) Eosinophil-deficient mice have impaired vascular reactivity. NE-induced constriction of arteries from WT and ΔdblGATA-1 mice (n = 15, data pooled from 3 experiments; ***P < 0.0001, two-way ANOVA) (ΔdblGATA-1 no PVAT (-•-), ΔdblGATA-1 PVAT (-ο-), WT no PVAT (-■-) and WT PVAT (-□-). (**c**) The PVAT-mediated anti-contractile response of eosinophil-deficient ΔdblGATA-1 mice (no PVAT (-•-) and PVAT (-ο-)) to NE-induced constriction is restored upon eosinophil-reconstitution (AdBac PVAT (-◊-) vs. AdBac no PVAT (-♦-) mean ± SEM; n = 10; P = 0.0023, two-way ANOVA). (**d**) Flow cytometry quantification of SMA^+^ contractile pericytes (mean ± SEM frequency pooled from 4 individual experiments; n = 6, P = NS, student’s t-test) showed no significant differences between WT (■) and ∆dblGATA-1 mice (-•-) in mesenteric adipose tissue, and (**e**) mesenteric adipose weight is similar in ∆dblGATA-1 and WT littermate control mice WT: n = 12 (■) and ∆dblGATA-1 (-•-) mice: n = 14 from 3 individual experiments; P = NS, student’s t-test). Flow cytometry analysis of digested mesenteric adipose tissue to identify (**f**) total and (**g**) RELMα^+^ CD206^+^ macrophages in mesenteric adipose tissue of WT (-■-) and ΔdblGATA-1 (-•-) mice. Graphs display the absolute numbers of total macrophages and RELMα^+^ CD206^+^ macrophages in the mesenteric adipose tissue (WT: n = 4 and ΔdblGATA-1: n = 7, P = NS, Student’s t-test).

To further investigate the role of eosinophils in vascular function we analyzed eosinophil-deficient ∆dblGATA-1 mice^13^. Flow cytometric analysis confirmed that eosinophils reside constitutively within mesenteric adipose tissue of wildtype (WT) mice (mean: 4.5% of all CD45^+^ cells, ± SD: 2.9%; n = 11), but are absent in ΔdblGATA-1 mice (Fig. 2a). As changes in perivascular adipose tissue has been shown to alter vascular function^14^ we examined the contractile and dilator response of small mesenteric arteries of ΔdblGATA-1 mice and their WT littermate controls by wire myography. In contrast with WT mice, in which there was a significant anti-contractile effect of PVAT (WT mice, PVAT vs. no PVAT: P < 0.0001, n = 15) (Fig. 2b), there was no PVAT-mediated anti-contractile effect to cumulative doses of NE in ΔdblGATA-1 mice (ΔdblGATA-1 mice, PVAT vs. no PVAT: P = NS, n = 15) (Fig. 2b). We additionally analyzed vessels with and without PVAT from IL-5 Tg mice that have excessive numbers of eosinophils and showed that IL-5 Tg arteries with or without PVAT responded in the same manner as WT arteries (Fig. S1a,b). Additionally, we observed that ΔdblGATA-1 mice had increased peripheral mean arterial blood pressure by an occlusion tail-cuff system (CODA) (P < 0.046; Fig. S1c) and elevated fasting blood glucose (P = 0.0106; Fig. S1d) compared with their littermate controls. Overall our data suggest that eosinophils are required for the PVAT-mediated regulation of vascular function.

#### Eosinophil reconstitution restores normal PVAT function

To confirm that changes in PVAT function in ΔdblGATA-1 mice were attributable to eosinophil-deficiency we performed eosinophil reconstitution (AdBac) by intravenous injection of purified eosinophils. Flow cytometric analysis confirmed that donor eosinophils localized to the adipose tissue of the mesenteric bed (Fig. 2a), as well as perigonadal adipose tissue and the small and large intestine (data not shown). Thirty days after eosinophil reconstitution functional evaluation of mesenteric vessels with and without PVAT to NE-stimulation further demonstrated that reconstitution of eosinophils completely restored the loss of PVAT-associated anti-contractile capacity (P = 0.0023; Fig. 2c) to levels comparable with WT controls (data not shown). The restoration of the anti-contractile capacity of PVAT following eosinophil reconstitution was independent of effects on smooth muscle, because neither AdBac nor ΔdblGATA-1 vessels devoid of PVAT had any significant differences in response to NE stimulation (Fig. 2c), demonstrating that the effect of eosinophils is mediated via PVAT. Moreover, a restoration of both peripheral MAP and blood glucose to WT levels was observed in ΔdblGATA-1 following reconstitution with eosinophils (MAP: WT vs. ΔdblGATA-1, P = 0.025; WT vs. AdBac: P = NS) (Fig. S1e; data not shown).

#### Eosinophils do not influence SMA^+^ pericytes or other immune populations

Further analysis of ΔdblGATA-1 mice showed no differences in the proportion of splenic or blood dendritic cells, neutrophils and monocytes compared with WT animals (Fig. S2b and c). In addition, we examined hemoglobulin (HGB) and hematocrit (HCT) levels and red blood cell (RBC) counts in ∆dblGATA-1 and age- and sex-matched littermate control mice, as these parameters were reported to be affected in eosinophil-deficient mice^13^. While the levels of RBCs (RBC (WT vs is ∆dblGATA-1): 9.7 ± 0.2 vs. 8.8 ± 0.2 /10^9^ cells/mL; P = 0.0338) were reduced in ∆dblGATA-1 mice, consistent with previous reports^13^, we detected no significant difference in the hemoglobulin (HGB: 140.6 ± 3.6 vs. 130.0 ± 3.3 g/L) or hematocrit (HCT: 0.51 ± 0.01 vs. 0.48 ± 0.01 L/L) levels.

Pericytes, including smooth muscle actin (SMA)^+^ contractile pericytes, are localized in the perivascular area, and are thought to be involved in vascular maintenance and function, including regulation of vascular constriction^15^,^16^. We therefore determined the frequency of pericytes in enzymatically digested mesenteric adipose tissue from WT and ΔdblGATA-1 mice. We detected no difference in the frequency of total or SMA^+^ contractile pericytes in ΔdblGATA-1 compared with WT mice (Fig. 2d and Fig. S2a). Similarly, we observed no difference in the total weight of the mesenteric adipose tissue between WT and ΔdblGATA-1 mice (Fig. 2e). Eosinophils have also been shown to affect macrophage functionality in adipose tissue^12^, and adipose tissue macrophages have in turn been shown to influence PVAT function^14^. To ascertain whether the altered vascular function in ΔdblGATA-1 mice could be attributed indirectly to a loss of eosinophil-mediated modulation of macrophage activation, we performed flow cytometry analysis of enzymatically digested mesenteric adipose tissue from ΔdblGATA-1 and WT mice. Macrophages were defined as live CD45^+^ F4/80^+^ CD11b^+^ CD64^+^ MHC-II^+^ Ly6G^−^ cells, and were further examined for expression of markers associated with alternatively activated macrophages (AAM), RELMα/FIZZ-1 and CD206^17^,^18^ (Fig. S2d). We detected no difference in the frequency and number of total CD45^+^ CD64^+^ CD11b^+^ MHC-II^+^ Ly6G^−^ macrophages in the mesenteric adipose tissue between WT and ∆dblGATA-1 mice (Fig. 2f) and, similarly, found no difference in the number of macrophages expressing RELMα and CD206, two markers associated with alternatively activated macrophages^17^,^18^ (defined as CD45^+^ F4/80^+^ CD64^+^ CD11b^+^ MHC-II^+^ Ly6G^−^ RELMα^+^ CD206^+^ cells) (Fig. 2g). Analysis of adipose tissue by qPCR also revealed no differences in levels of the AAM markers *arginase-1, fizz-1* and *ym-1* between ΔdblGATA-1 and WT mice (Fig. S2e–g). Taken together, these data suggest that alterations in pericyte or macrophage composition, or adipose tissue hypertrophy, do not explain the impaired vascular reactivity in ΔdblGATA-1 mice, indicating that eosinophils may be directly involved in the regulation of PVAT function rather than acting via other cell populations.

### Eosinophils influence vascular reactivity via PVAT-mediated release of soluble mediators

**Figure 3:**
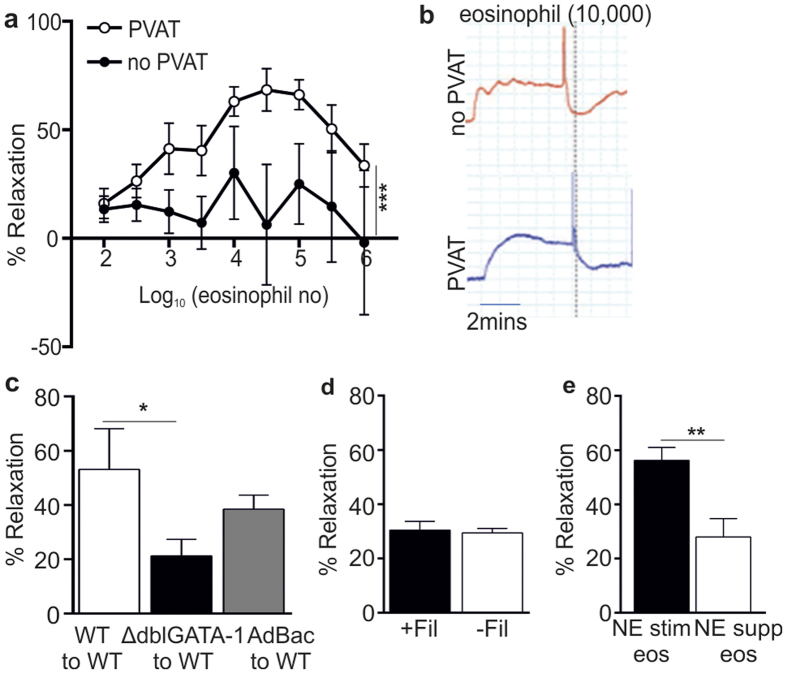
Exogenous application of purified eosinophils induces vessel relaxation. Isolated splenic eosinophils were added to mesenteric arteries preconstricted with norepinephrine (10^−5^ M). Relaxation was measured by wire myography. **(a)** Exogenous application of eosinophils induced dose-dependent relaxation of ΔdblGATA-1 arteries in the presence (-ο-) but not absence (-•-) of PVAT (n = 8, pooled from 8 individual experiments; ***P < 0.0001, two-way ANOVA). **(b)** Trace representative of a pre-constricted ΔdblGATA-1 artery ± PVAT responding to 10,000 eosinophils. Note the rapid drop in constriction following addition of eosinophils (dotted line). **(c)** Solution transfer experiments to assess generation of a transferable factor that mediates relaxation (n = 10, pooled from 2 experiments; *P = 0.049, Student’s t-test. **(d)** Vessel relaxation in response to application of NE-stimulated eosinophils (−Fil) or filtered supernatant from NE-stimulated eosinophils (+Fil), by passing through a 0.2 μm filter to retain eosinophils, to pre-constricted WT mesenteric arteries in the presence of PVAT. **(e)** Addition of NE-stimulated (10^−5^ M) eosinophil supernatant or unstimulated eosinophil supernatant supplemented with NE (10^−5^ M) to pre-constricted ΔdblGATA-1 arteries in the presence of PVAT (n = 6; **P = 0.0068, student’s t-test).

To determine whether eosinophils directly influence PVAT function to modulate downstream vascular reactivity, purified eosinophils were added to pre-constricted (1 × 10^−5^ M NE) mesenteric arteries ± PVAT from ΔdblGATA-1 mice. The addition of purified eosinophils (100 to 30,000 cells) was associated with a significantly greater dose dependent relaxation in vessels with PVAT compared with vessels without PVAT (P < 0.0001) (Fig. 3a). Relaxation occurred within 30 seconds of addition of eosinophils to the organ bath, demonstrating a rapid effect of eosinophils at both the vascular and perivascular level *in vitro* (Fig. 3b). In contrast, addition of 10,000 purified macrophages had no PVAT-dependent relaxant effect (data not shown). Indeed, relaxation was attenuated at higher eosinophil concentrations (100,000 to 1,000,000 eosinophils), which may be associated with concentrations of eosinophils beyond normal physiological levels resulting in the production of oxygen free radicals^19^ and subsequent vascular constriction^20^.

To establish whether eosinophils promoted the release of a soluble anti-contractile factor(s) from PVAT, organ bath solution transfer experiments were performed. Total organ bath solution from pre-constricted (1 × 10^−5^ M NE) small mesenteric arteries was transferred from donor arteries (with PVAT) to recipient arteries (without PVAT). These experiments demonstrated that healthy WT PVAT secretes a soluble relaxing factor (Fig. 3c)^21^. However, relaxation was impaired when solution from ΔdblGATA-1 donor arteries was transferred to WT recipient arteries (ΔdblGATA-1 to WT, P = 0.049; Fig. 3c). Furthermore, transfer studies between WT donor arteries and ΔdblGATA-1 recipient arteries demonstrated reduced relaxation compared with WT control solution transfer (data not shown). Importantly, *in vivo* reconstitution with eosinophils restored normal PVAT function. Thus, vessel relaxation was restored to the same levels as WT to WT transfer in both donor and recipient AdBac transfers with WT vessels (Fig. 3c and data not shown). These data implicate a role for eosinophils in mediating the secretion of a soluble relaxant factor(s) by PVAT.

To determine whether the relaxant effect of eosinophils was due to the release of eosinophil-derived soluble factors or via direct eosinophil interaction with PVAT, relaxation of C57BL/6 mesenteric arteries + PVAT was compared following application of 10,000 NE-stimulated eosinophils or the filtered supernatant from 10,000 NE-stimulated eosinophils. The filtered eosinophil supernatant was capable of inducing relaxation of mesenteric vessels to the same extent as the addition of eosinophils (Fig. 3d). Furthermore, to ensure that our results were not due to an effect of NE-dilution when eosinophils are added to NE-preconstricted arteries, eosinophils were added in a buffer containing 1 × 10^−5^ M NE. However, as eosinophils express adrenergic receptors^22^ and could potentially respond to NE; we examined the effect of NE-treatment on eosinophils. Supernatant from 10,000 eosinophils stimulated with 1 × 10^−5^ M NE or supernatant from unstimulated eosinophils supplemented with 1 × 10^−5^ M NE was added to ΔdblGATA-1 arteries with PVAT. The NE-supplemented supernatant from unstimulated eosinophils induced a significantly reduced relaxation of the mesenteric vessels in comparison with NE-stimulated eosinophils (P = 0.0068; Fig. 3e), suggesting that eosinophil stimulation with NE is important in mediating eosinophil-induced vascular relaxation. Collectively, these data implicate a soluble factor released from NE-stimulated eosinophils, rather than cell to cell contact, in mediating eosinophil-induced PVAT function.

### Eosinophils act via adrenergic stimulation of nitric oxide and adiponectin release

**Figure 4:**
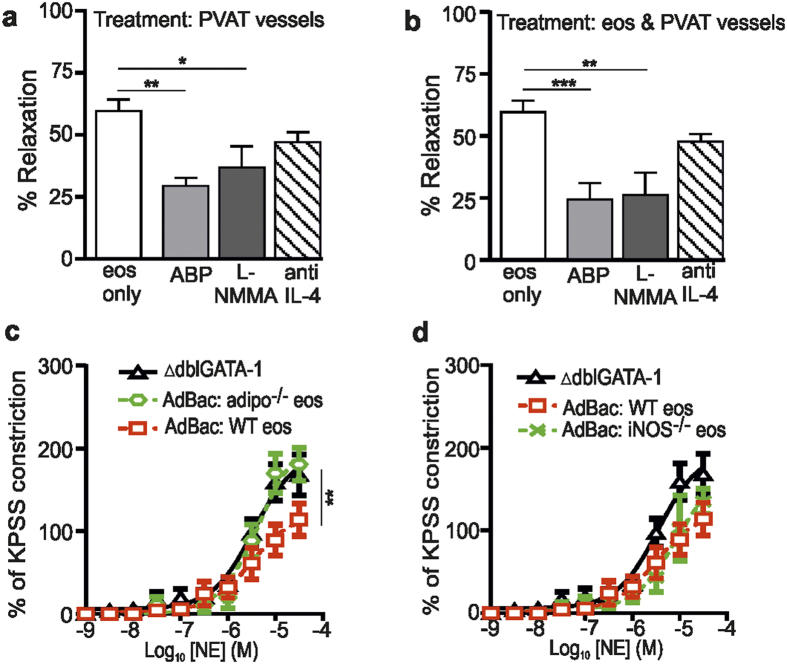
Eosinophils induce adiponectin- and NO-dependent PVAT-induced vessel relaxation. The functional role of adiponectin and NO were investigated by wire myograph analysis of mesenteric vessels of ΔdblGATA-1 mice using pharmacological tools and in eosinophil-reconstituted ΔdblGATA-1 mice. **(a,b)** Vessel relaxation in response to exogenous application of 10,000 NE-stimulated eosinophils (eos) to preconstricted ΔdblGATA-1 mesenteric arteries + PVAT following incubation of **(a)** PVAT (L-NMMA, anti-IL-4: n = 9, ABP: n = 8; *P < 0.05 and **P < 0.01, one-way ANOVA with post hoc Dunnett’s), or **(b)** PVAT and eosinophils (L-NMMA, ABP, anti-IL-4: n = 9; **P < 0.01, ***P < 0.001, one-way ANOVA with post hoc Dunnett’s). **(c,d)** The PVAT anti-contractile effect of NE constricted arteries from ΔdblGATA-1 mice reconstituted with **(c)** adiponectin^−/−^ (-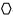-), **(c,d)** WT (-□-), or **(d)** iNOS^−/−^ (-×-) eosinophils (adipo^−/−^ vs WT: n = 4, **P = 0.0085, two-way ANOVA; iNOS^−/−^ vs WT: n = 5; P = NS, two-way ANOVA).

Our previous data have shown that adiponectin^4^,^21^ and nitric oxide^23^ are important in mediating normal PVAT relaxing function. Furthermore, IL-4 is a previously recognised eosinophil-derived effector molecule. Therefore, we tested the functional role of these putative mediators on the vascular reactivity of vessels in response to the addition of 10,000 eosinophils. To this end, mesenteric vessels + PVAT isolated from ∆dblGATA-1 mice and/or purified eosinophils were incubated with the NO-signalling inhibitor L-NMMA (1 × 10^−5^ M)^23^, adiponectin blocking peptide (ABP; a soluble fragment of the Type-1 adiponectin receptor; 5 μg/mL)^4^ or anti-IL-4 antibody (0.4 μg/mL) for 30 minutes before pre-constriction of the vessels with 10^−5^ M NE and addition of 10,000 stimulated eosinophils (1 × 10^−5^ M NE). Incubation of vessel + PVAT alone with ABP and L-NMMA, but not anti-IL-4, reduced eosinophil-induced relaxation (ABP vs control, P < 0.01; L-NMMA vs control, P < 0.05; Fig. 4a), whereas incubation of eosinophils alone with the inhibitors had no effect on relaxation (data not shown). There was also a significant reduction in relaxation observed following incubation of both vessels and eosinophils with L-NMMA and ABP, but not anti-IL-4, compared with the addition of stimulated eosinophils alone (L-NMMA vs. control: P < 0.01; ABP vs. control: P < 0.001; Fig. 4b). In contrast, incubation of eosinophils, vessel or eosinophils and vessels with the inhibitors in the absence of PVAT failed to alter relaxation of the arteries to NE-stimulated eosinophils (data not shown). Similarly, addition of D-NMMA, IgG or a control peptide (the negative controls for L-NMMA, anti-IL-4 and ABP, respectively) to vessels + PVAT had no effect on PVAT function (data not shown).

To examine if eosinophils could act as source of NO or adiponectin, we performed eosinophil reconstitution experiments using bone marrow-derived eosinophils from WT, iNOS^−/−^ or adiponectin^−/−^ mice. PVAT vessels from reconstituted mice showed a loss of anti-contractile function following reconstitution with adiponectin^−/−^ eosinophils, but not iNOS^−/−^ or WT eosinophils (adipo^−/−^ vs WT: P = 0.0085; Fig. 4c and d). Collectively, these data suggest that adiponectin and NO signalling are critical components of the regulation of vascular activity, and further, that eosinophils may act as a source of adiponectin, as well as inducing the release of downstream adiponectin from PVAT, resulting in relaxation of vessels via NO-dependent mechanisms.

**Figure 5:**
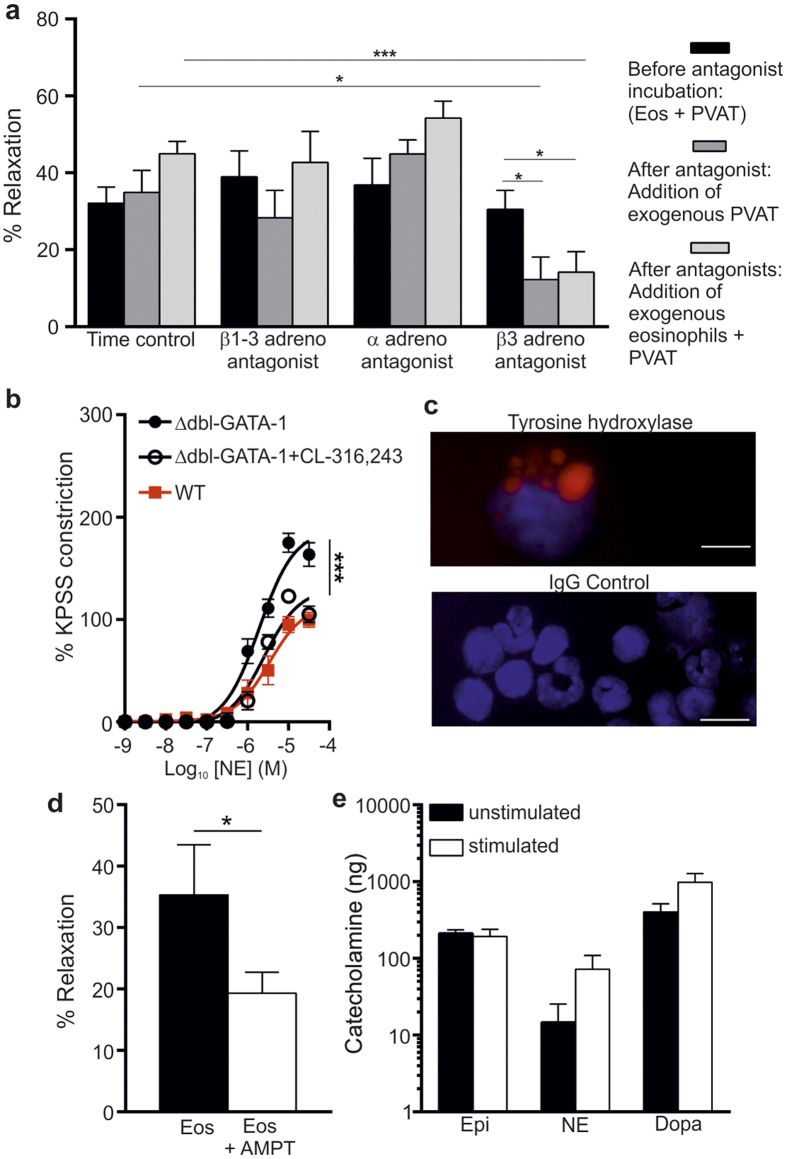
Eosinophils stimulate β3 adrenoreceptors through the release of soluble factors. (**a**) Samples of PVAT from WT mice were incubated with or without antagonists of β1-β3 (propranolol 1 μM) and, α1 (phentolamine 0.1 μM,) or β3 (SR-592,30 A 0.3 μM) adrenoreceptors and the anti-contractile response of NE-constricted arteries assessed by wire myography (β3 adrenoreceptor antagonist vs. time control: ***P < 0.001, one-way ANOVA; β3 adrenoreceptor antagonist vs. vessel control, n = 6; *P < 0.05, one-way ANOVA). (**b**) The β3 adrenoreceptor agonist (CL-316,243) was added to ΔdblGATA-1 arteries with PVAT and contractile responses compared with WT (P < 0.001, n = 5, one-way ANOVA with post hoc Dunnett’s). (**c**) Immunocytochemical analysis for tyrosine hydroxylase in purified and cytospun splenic eosinophils and IgG control staining. (**d**) Eosinophils were incubated with or without a tyrosine hydroxylase inhibitor (AMPT) and the vascular reactivity of the vessels in response to addition of 10,000 eosinophils tested (*p = 0.0472, n = 5, t-test). (**e)** Levels of epinephrine, norepinephrine and dopamine were measured from unstimulated and stimulated eosinophils (n = 4) by ELISA.

Given the rapid effect on PVAT function following addition of eosinophils (Fig. 3b), we hypothesized that eosinophils may directly exert their effect via adrenoreceptors. To explore this idea, we incubated mesenteric vessels + PVAT with eosinophils in the presence or absence of antagonists of β1-β3, α1 or β3 adrenoreceptors. After addition of antagonists against β1-β3 or α1 adrenoreceptors, there was no inhibition of relaxation in the presence of exogenous PVAT or exogenous PVAT with eosinophils (Fig. 5a). In contrast, a specific β3 adrenoreceptor antagonist (SR-592,30 A)^24^ significantly reduced relaxation in the presence of exogenous PVAT or exogenous PVAT with eosinophils (β3 adrenoreceptor antagonist vs. time control, P < 0.001 and β3 adrenoreceptor antagonist vs. vessel control, P < 0.05; Fig. 5a). Moreover, the direct addition of the β3 adrenoreceptor agonist (CL-316,243)^25^ to ΔdblGATA-1 arteries with PVAT resulted in a significant relaxation of these vessels in comparison to control ΔdblGATA-1 arteries with PVAT (P < 0.001; Fig. 5b), suggesting that this elicits the same response as the addition of eosinophils. The only known agonists of β3 adrenoreceptors are catecholamines, the production of which is critically dependent on the enzyme tyrosine hydroxylase^26^. As our data demonstrated that eosinophils secrete a soluble factor that mediates a relaxant effect on PVAT, we hypothesised that eosinophils may be a source of catecholamines. Immunocytochemical staining revealed high expression of tyrosine hydroxylase in eosinophil granules (Fig. 5c). To examine the functional significance of this, 10,000 eosinophils were incubated with or without a tyrosine hydroxylase inhibitor (AMPT). Addition of eosinophils pre-incubated with AMPT to mesenteric vessels + PVAT isolated from ∆dblGATA-1 mice resulted in a significantly decreased relaxation compared to untreated eosinophils (P = 0.0472; Fig. 5d). Finally, we demonstrated that eosinophils are able to constitutively produce epinephrine, NE and dopamine, and with a trend towards increased NE and dopamine secretion following activation of eosinophils with IL-5 and eotaxin (Fig. 5e). Together these data demonstrate that PVAT functionality depends on tyrosine hydroxylase-dependent eosinophil production of catecholamines that signal via PVAT-located β3 adrenoreceptors.

## Discussion

The anti-contractile function of perivascular adipose tissue (PVAT) is lost in obesity, a disorder with an underlying immunological component^9^,^27^ that has been linked with increased peripheral vascular resistance and elevated peripheral blood pressure^5^,^7^. While a role of eosinophils in regulation of these events have not previously been recognized, our data identify mechanisms by which obesity-induced alterations to the eosinophil population may perturb the influence from PVAT on small arteries and the physiological consequences thereof^4^,^6^. Thus, for the first time, we demonstrate that eosinophils play a central role in the release of vasorelaxing factors from healthy PVAT, via the release of eosinophil-derived catecholamines, to mediate adipocyte-localised β3 adrenoceptor activation and downstream adiponectin- and nitric oxide-signalling (Fig. 5 **–** Fig. Sup. 1). Loss of PVAT function was also associated with increased arterial tone, with a potential role of eosinophils in the regulation of these processes being further supported by our observations that hypertensive eosinophil-deficient mice recovered upon eosinophil reconstitution. Furthermore, the loss of a vasodilatory paracrine effect from adipose tissue has been suggested to limit downstream microcirculatory nutritive flow^6^, thereby contributing to insulin resistance and metabolic dysregulation. It is clear from these studies that eosinophils play a vital role in sustaining normal PVAT function in health to maintain adipose tissue homeostasis, which have important downstream physiological consequences.

Previous work to understand the mechanisms responsible for the anti-contractile effect of PVAT have focused attention on the macrophage^14^, whilst other leukocyte populations have largely been ignored. Eosinophils have previously been shown to be indirectly involved in regulation of events in adipose tissue by controlling adipose tissue macrophage functionality^12^, which in turn can influence PVAT function in inflammation^14^. For example in obesity, factors such as tissue hypoxia and aldosterone signaling are thought to alter macrophage activation, which subsequently impacts on PVAT anti-contractile function, manifesting in hypertension^14^. Macrophages have also been shown to play a role in beiging of fat and thermoregulation^28^. A recent report implicated eosinophils in regulation of the polarization of white adipose tissue macrophages to an AAM phenotype. Thus, Wu *et al*.^12^ detected a decrease in a subpopulation of YFP^+^ macrophages expressing high levels of CD11b and F4/80 in the perigonadal adipose tissue of ΔdblGATA-1 BALB/c mice crossed with mice expressing YFP under the arginase-1 promoter. In apparent contrast to these results, we detected no change in the frequency of total or AAM in the mesenteric adipose tissue of ΔdblGATA-1 compared with WT mice. Although the reason for this discrepancy is not entirely clear, we speculate that differences in the source of adipose tissue or analysis may underlie the difference. Thus, while Wu *et al*. examined a population of GFP-positive cells among a population of F4/80^hi^ CD11b^hi^ perigonadal adipose tissue macrophages in arginase-reporter mice^12^, we analyzed expression of RELMα and CD206 on total CD11b^+^ CD64^+^ mesenteric adipose tissue macrophages. Consistent with our flow cytometry analysis, qPCR analysis of mesenteric adipose tissue from WT or ΔdblGATA-1 mice revealed no differences in the expression of *arginase-1, fizz-1* or *ym-1*. Finally, we also addressed a potential role of IL-4 in PVAT function, as IL-4 secretion from eosinophils has been proposed to be a key mechanism by which eosinophils sustain AAM and regulate glucose homeostasis^12^. However, neutralization of IL-4 had no impact on vessel reactivity in our system. Although we cannot exclude the possibility that macrophages may indirectly contribute to PVAT function, the current study demonstrate that eosinophils contribute to regulation of vascular function directly, by release of catecholamines that act rapidly via PVAT to promote vessel relaxation. This appears to be a unique function of eosinophils, and cannot be mediated by other immune cells, including macrophages. Taken together we conclude that eosinophils exert an effect on healthy PVAT function directly, and independently of other immune cell populations.

Adipocytes and eosinophils are known to secrete a number of adipokines and cytokines, which influence downstream biological responses^10^,^12^,^29^. The balance of pro- and anti-inflammatory adipokines is important in mediating the anti-contractile capacity, energy homeostasis and inflammatory status of PVAT. Ours and others’ previous data in humans and rodent species (rats and mice) have shown that adiponectin^4^,^21^ and nitric oxide signaling^23^,^30^ are important in mediating normal PVAT function. Thus, adiponectin-deficient mice demonstrate a loss of PVAT function in response to adrenergic stimulation^21^ and have been shown to be hypertensive^31^. Furthermore, healthy PVAT-induced vascular relaxation is attenuated by inhibition of NO, and NO signaling is dysregulated in a rat model of the metabolic syndrome associated with enhanced vasoconstriction to norepinephrine^30^. Indeed in line with this, we confirmed that adiponectin and NO signaling were essential for the anti-contractile function of PVAT also in mice, and that eosinophils were necessary for promoting the adiponectin- and NO-dependent anti-contractile function of PVAT. Furthermore, to examine if eosinophils could act as a direct source of NO or adiponectin, we performed *in vivo* reconstitution experiments using eosinophils generated from WT, iNOS^−/−^ or adiponectin^−/−^ mice. Interestingly, our experiments revealed that eosinophil-derived adiponectin, but not NO, contributed to the anti-contractile capacity of PVAT. Thus, our data suggest that eosinophils may act as a source of adiponectin as well as inducing the release of downstream adiponectin from PVAT, resulting in relaxation of vessels via NO-dependent mechanisms.

The eosinophil cytoplasm contains numerous preformed granules containing a variety of effector molecules; therefore we hypothesized that eosinophils may act on PVAT via secreted factors. In support of this hypothesis, addition of filtered eosinophil supernatant to vessels +PVAT mimicked the relaxant effect of eosinophil addition. Moreover, as the eosinophil-dependent effect was so rapid, we focused our studies on adrenoreceptor-mediated activation of PVAT rather than genomic manifestations of eosinophil-deficiency. Addition of a panel of adrenoreceptor inhibitors in our experimental model demonstrated that the vessel-relaxing interaction of eosinophils with PVAT occurred via the β3 adrenoreceptor, supporting a previous study that identified a role for β3 adrenoreceptors in mediating the adipocyte-derived hyperpolarizing factor (ADRF)-induced hyperpolarization of smooth muscle^25^. Catecholamines are the only known activators of the β3 adrenoreceptor, and depend on the enzyme tyrosine hydroxylase for their production^26^. Immunocytochemical analysis of purified eosinophils demonstrated that eosinophils indeed express this enzyme, which was necessary for the eosinophil-mediated effect on vascular reactivity. Furthermore, we showed that eosinophils constitutively secrete catecholamines. To our knowledge, this is the first time that eosinophils have been reported to express tyrosine hydroxylase and secrete catecholamines.

In conclusion, our data demonstrate for the first time a direct role for eosinophils in regulation of adipose tissue functionality, further emphasizing the surprising contribution of eosinophils to physiological processes beyond immune function. We have demonstrated that eosinophils play a crucial role in mediating normal PVAT function, the dysfunction of which is associated with conditions such as hypertension and type 2 diabetes. Thus, eosinophil-deficiency within adipose tissue led to physiological consequences on vascular reactivity, independent of other immune cell populations. The effects on PVAT and vascular function are mediated via regulation of NO-signaling and release of adiponectin, following secretion of catecholamines that promote β3 adrenoreceptor-dependent PVAT anti-contractile function (Fig. 5, Supplement 1). We propose the following model for regulation of vessel reactivity by eosinophils (see also Fig. 5, Supplement 1): Eosinophils release catecholamines that stimulate adipocytes by signaling via beta 3-adrenoreceptors. In response to stimulation, adipocytes produce adiponectin and NO, which subsequently act on the vascular smooth muscle to mediate vessel relaxation. Taken together, our data identifies eosinophils as novel targets for the development of therapies for obesity and related cardiovascular complications.

## Methods

∆dblGATA-1, IL-5 Tg and littermate control mouse details, standard experimental procedures, and more detailed descriptions of protocols and analyses are presented in the online Supplemental Experimental Procedures-section. The IL5 Transgenics were from the laboratories of Drs Nancy and Jamie Lee.

### Eosinophil purification and transfer

Eosinophils were purified from IL-5 Tg mice by negative selection using MACS columns or by cell sorting, and were approximately 95% pure irrespective of purification method (data not shown), as assessed by flow cytometry of CD11b^+^ SiglecF^+^ SSC^high^ cells. For adoptive transfers, eosinophils were resuspended in sterile phosphate buffered saline (PBS), and 100–150 million cells injected intravenously, and recipient mice analysed 30 days after transfer. Bone marrow-derived eosinophils were grown as described previously^32^. After 12–14 days in culture the cells were >90% pure as determined by flow cytometry analysis; cells were resuspended in sterile PBS, and 1–3 million cells injected intravenously per mouse, and recipient mice analysed five days after transfer.

### Adipose tissue digest

For isolation of the adipose stromal vascular fraction (SVF), white mesenteric adipose tissue was finely minced and incubated in medium containing 1 mg/mL type I collagenase and 30 μg/mL DNase I at 37 °C for 45 min with magnetic stirring at 350 rpm. The resulting cell suspension was spun to separate floating adipocytes from the SVF pellet, and passed through 40 μm filters to generate a single-cell suspension. Leukocytes were further enriched by density centrifugation using Percoll, followed by phenotypic and quantitative analysis by flow cytometry.

### Pharmacological assessment of vascular reactivity by wire myography

The mesenteric bed was removed and placed in ice-cold physiological salt solution (PSS); first-order arteries were identified and dissected clean of PVAT or left with surrounding PVAT intact as indicated. Vessels were mounted on a wire myograph, equilibrated, normalised and viability assessed as previously described^4^,^23^,^33^. Vascular contractility was assessed in vessels to increasing concentrations of norepinephrine (NE).

Solution transfer experiments were performed between wildtype (WT), IL-5 Tg, ∆dblGATA-1 and eosinophil-reconstituted ∆dblGATA-1 (AdBac) mice. Arteries were preconstricted with NE and a stable constriction established; next, total myograph bath solution (6 mLs) taken from donor arteries with PVAT were used to replace the solution from a recipient artery devoid of PVAT.

In order to investigate the direct effect of eosinophils on vascular contractility, the exogenous addition of (1 × 10^2^ to 1 × 10^6^) eosinophils to preconstricted mesenteric arteries (1 × 10^−5^ M NE) from ΔdblGATA-1 mice was assessed in the presence and absence of PVAT. To establish whether eosinophils interacted directly or indirectly, the supernatant from NE-stimulated eosinophils was added to preconstricted vessels.

Adiponectin blocking peptide, L-NMMA, anti-IL-4, propranolol and SR-592,30 A or appropriate controls were incubated for 30 minutes at 37 °C with either eosinophils only, arteries ± PVAT, or both eosinophils and arteries (±PVAT). Both eosinophils and vessels were treated with NA (1 × 10^−5^ M) before addition of eosinophils to the vessels. The response to eosinophil addition was measured as a percentage relaxation.

### Statistics

Differences in response to NE were expressed as a percentage of constriction to KPSS^4^,^14^,^21^,^23^; functional differences between groups were examined for statistical significance using two-way ANOVA when appropriate, with the Bonferroni post-hoc test or one-way ANOVA with post hoc Dunnett’s test when appropriate. All other statistical analysis was performed by student’s t-test, unless stated. P-values below 0.05 were considered significant (*P < 0.05, **P < 0.01, ***P < 0.001). Data are expressed as mean ± SEM unless otherwise stated. GraphPad Prism, version 6.00 for Windows was used for data analysis.

### Ethical approval

Procedures were performed in accordance with the United Kingdom Animals (Scientific Procedures) Act of 1986, and conformed to the Directive 2010/63/EY of the European Parliament. Ethical permission was obtained from the Local Ethical Committee at Lund University, Sweden, and the University of Manchester Animal Welfare and Ethical Review Board and performed under a Home Office approved grant.

## Additional Information

**How to cite this article:** Withers, S. B. *et al*. Eosinophils are key regulators of perivascular adipose tissue and vascular functionality. *Sci. Rep.* **7**, 44571; doi: 10.1038/srep44571 (2017).

**Publisher's note:** Springer Nature remains neutral with regard to jurisdictional claims in published maps and institutional affiliations.

## Supplementary Material

Supplementary Information
