# Supplementary material for: Eosinophils are key regulators of perivascular adipose tissue and vascular functionality
Source: Sci Rep. 2017 Mar 17;7:44571. doi: 10.1038/srep44571 (PMC5356000; doi:10.1038/srep44571)
Supplement: Supplementary Information [file srep44571-s1.pdf]

Online Supplement

Title: Eosinophils are key regulators of perivascular adipose tissue and vascular functionality.

Authors: Sarah B. Withers[†1, 6], Ruth Forman[†,2], Selene Meza-Perez[3,5], Daniel Sorobetea[3], Kasia Sitnik[4], Thomas Hopwood[2], Catherine B. Lawrence[2], William W. Agace[3, 4], Kathryn J. Else[2], Anthony M. Heagerty[§, 1], Marcus Svensson-Frej[§, 3], and Sheena M. Cruickshank[§, 2,*]

Detailed Methods:

Study Design

The hypothesis of this work was that eosinophils, a major cell component of mesenteric adipose tissue, would have an effect on PVAT function. Thus, initial experiments aimed at defining such a role, with further work designed to unravelling the mechanisms and testing whether the effect of eosinophils was direct or indirect.

Animal numbers in each experiment were based on the minimum required per group to show statistical significance. From our previous experience this is typically 5-8 per group. Outcome parameters were in the form of numerical data and most studies were based on a 'fixed effects' model that compares either one, or several, treatment(s) to control. Data were normally distributed, and no data have been excluded. Mice were selected based on genotype, sex and age, and all mice were matched with littermate controls; only males of the ages 8-14 weeks were used in the study. Mice were randomly assigned to treatment groups from different cages and litters: the records were all kept on an excel spreadsheet that contained no information about cage location or cage number, and mice were randomly selected from this list and allocated to the appropriate treatment group. Blood pressure and glucose readings were recorded in a blinded manner, by allocating a number to each mouse. After all readings were completed, mice were unblinded and their genotype identified.

Endpoints *in vivo* were defined based on ability to detect eosinophils. Initial experiments used long-term (30 days) reconstitution of mice with eosinophils, but subsequent experiments revealed the data obtained 5d post-reconstitution was comparable to that from d30 post-reconstitution and therefore

used a shorter time frame. Similarly, the original number of cells for the reconstitution was based on published methods, but subsequent optimisation experiments revealed that identical results were achieved using a lower cell numbers. There were no anticipated adverse effects and none were observed. Most experiments were performed at least twice on at least 4-6 mice per group, as stated on the corresponding figures. Units were mice for *in vivo* experiments and for *ex vivo* analysis.

Animals

ΔdblGATA-1 mice were a kind gift from Professor Avery August, Pennsylvania State University, USA. ΔdblGATA-1 mice were bred with C57BL/6 mice and F1 hybrid offspring mated with their siblings to produce the F2 generation, this F2 generation was then maintained by intercrossing heterozygous females and hemizygous males. All experimental ΔdblGATA-1 and WT animals used are littermate F2 mice. IL-5 transgenic (IL-5Tg) mice were from Professor James Lee (Mayo Clinic, USA) and were maintained by crossing heterozygous mice with C57BL/6 mice. Where indicated, obese C57BL/6 DIO mice (HFD) (The Jackson Laboratory, USA) were maintained from weaning on a high-fat diet (composition: protein: 20%, carbohydrate: 20%, fat: 60%; Research Diets, Inc., D12492)[1] and used at 12 weeks of age. Bones from iNOS$^{-/-}$ mice (generous gift from Dr Caroline Chadwick, University of Birmingham) and adiponectin$^{-/-}$ mice (generous gift from Dr Yu Wang, Hong Kong University) were used to generate bone marrow-derived eosinophils [2] . Mice were culled by CO2 followed by cervical dislocation. Animal numbers were based on the minimum required per group to show statistical significance; from our previous experience this is typically 4-8 mice per group. Outcome parameters were in the form of numerical data and most studies were based on a 'fixed effects' model that compares either one, or several, treatment(s) to control. Mice were selected based on genotype, sex and age, and all mice were matched with littermate controls.

Physiological parameters

Systemic Arterial Blood pressure

Systolic and diastolic blood pressure was measured non-invasively, by determining the tail blood volume with a volume pressure recording sensor and an occlusion tail-cuff (CODA System, Kent Scientific, Torrington, CT)[3, 4]. Mice were subject to equilibration readings before measurement of actual blood pressure from 8-10 readings per mouse. Measurements that did not satisfy the volume occlusion specifications or were deemed too short were excluded from analysis. Blood pressure was measured at the same time of the day for each experimental group and recorded in a blinded manner, by allocating a number to each mouse. After all readings were completed, mice were unblinded and their group belongings identified.

Blood glucose

Mice were fasted overnight for 16 hours and blood glucose was measured from tail-vein samples using a hand-held glucose monitor (Accu-Check Aviva, Roche, UK)

Cell isolation

Eosinophil purification

Eosinophils were purified from IL-5 Tg mice by negative selection using MACS columns or by cell sorting, and were approximately 90-96% or >95% pure for *in vivo* and *in vitro* experiments irrespective of purification method, respectively, as assessed by flow cytometry of $CD11b^{+}$ $SiglecF^{+}$ SSC^{high} cells. For adoptive transfers, eosinophils were resuspended in sterile PBS, and 100-150 million cells injected intravenously. Bone marrow-derived eosinophils were grown as described previously [2]. After 12-14 days in culture the cells were >90% pure as determined by flow cytometry analysis; cells were resuspended in sterile PBS, and 1-3 million cells injected intravenously per mouse, with analysis five days after transfer.

Catecholamine ELISA

Splenic eosinophils were isolated from IL-5Tg mice and purified as described above. Eosinophils were purified using MACS columns and stimulated with eotaxin (200ng/ml; R&D systems), IL-5 (400U/ml; R&D systems) and cytochalsin B (50µg/ml, Sigma-Aldrich) for 2hours. Catecholamine levels were determined using the Alpco Tri-CAT ELISA according to manufacturer instructions.

Characterisation of adipose tissue cell populations

Adipose tissue digest

For isolation of the adipose stromal vascular fraction (SVF), white mesenteric adipose tissue was finely minced and incubated in medium (DMEM (Life technologies), 25mM HEPES (Life technologies), 4% (w/v) BSA (Sigma-Aldrich)) containing 1mg/mL type I collagenase (Life technologies) and 30µg/mL DNase I (Roche diagnostics) at 37°C for 45 min with magnetic stirring at 350rpm. The resulting cell suspension was spun at 1000 ×g for 10 min to separate floating adipocytes from the SVF pellet and the isolated SVF resuspended in MACS buffer (PBS (Life technologies), 2% FCS (Sigma-Aldrich), 2mM EDTA (Sigma-Aldrich), 0.05% NaN_3 (Sigma-Aldrich)) and passed through 40 µm filters to generate a single-cell suspension. Leukocytes were enriched by density centrifugation using 40 and 70% Percoll (GE Healthcare) at 600 ×g for 20 min at room temperature. The layer over the 70% Percoll was harvested, washed, and resuspended in MACS buffer.

Flow cytometry analysis

Single cell suspensions were incubated with 5µg/mL anti-CD16/CD32 antibody (clone 2.4G2; BD Biosciences) in MACS buffer for 20 min on ice to block Fc receptors, followed by staining with

indicated antibodies for 20 min on ice, and data acquisition on a LSRII flow cytometer (BD Biosciences). The following monoclonal antibodies and reagents were used: anti-Siglec-F (E50-2440; BD Biosciences), anti-CD11c (N418; eBiosciences), anti-F4/80 (BM8; eBiosciences), anti-CD11b (M1/70; BioLegend), anti-CD45 (104; BioLegend), anti-MHC-II (M5/114.15.2; BioLegend), anti-CD63 (NVG-2; BioLegend), anti-CD206 (C068C2; BioLegend), anti-CD64 (X54-5/7.1; BioLegend), anti-SMA (1A4; Abcam), and-CD31 (390; eBiosciences), anti-PDGFRb (APB5; eBiosciences), and anti-gp38 (8.1.1; BioLegend). LIVE/DEAD Fixable Violet Dead reagent (Life Technologies) was used to distinguish dead cells. For intracellular staining for RELMα, cells were surface-labelled as described above, followed by fixation/permeabilization using the foxp3 staining buffer set (eBioscience). Cells were labelled with polyclonal rabbit-anti-mouse RELMα (Peprotech), followed by incubation with Zenon anti-rabbit secondary reagent (Life technologies).

Pharmacological assessment of vascular reactivity by wire myography

The mesenteric bed was removed and placed in ice-cold PSS (119mM NaCl, 4.7mM KCl, 25mM $NaHCO_3$, 1.17mM KH_2PO_4, 1.17mM $MgSO_4$, 0.026mM EDTA, 1.6mM $CaCl_2$ and 5.5mM glucose; Sigma-Aldrich); first-order arteries were identified (diameter: 200μM, length: 0.5-1mm) and one part was dissected clean of PVAT, whereas the other part was left with surrounding PVAT intact. Vessels were mounted on two 40μm tungsten wires in a wire myograph (Danish MyoTech, Aarhus, Denmark). Vessels were incubated in PSS at 37°C and bubbled with 95% air/5% CO_2 to saturate the vessel bath whilst maintaining pH ≈7.4, as previously described [5, 6]. Following equilibration for 30 min[7], vessel wall tension and diameter were normalised using a standardised procedure according to the Laplace equation and stabilised for a further 30 min [7]. Constriction of arteries with high-potassium PSS (60 mM) (KPSS) was used to assess arterial viability and addition of 1×10^{-5} M acetylcholine (ACh) (Sigma-Aldrich) determined endothelial integrity of the arteries; arteries exhibiting a relaxation of >25% were deemed to have impaired endothelium and excluded from the study. Vascular contractility was assessed in vessels with and without PVAT to increasing concentrations of NE ($1x10^{-9}$ to $3x10^{-5}$M) (Sigma-Aldrich). Constriction with KPSS was performed after each NE concentration response curve, acting as an internal time control and reference of contractility for each response curve [5, 8, 9].

Solution transfer experiments

Solution transfer experiments were employed to study the role of eosinophils in the release of relaxing factors from PVAT or in mediating the downstream vascular response. Experiments were performed between WT, IL-5 Tg, ΔdblGATA-1 and AdBac mice. Arteries with and without PVAT were preconstricted with NE (1×10^{-5}M) and stable constriction established for 3 min [10]. Total myograph bath solution (6mLs) was taken from donor arteries with PVAT and used to replace the solution from a recipient artery devoid of PVAT (Supplementary Fig. 3). Change in tension was calculated as

percentage relaxation of the constricted artery and compared with relaxation following solution transfer between WT-donor to WT-recipient transfer.

Investigating the direct effect of eosinophils on vascular reactivity

In order to investigate the direct effect of eosinophils on vascular contractility, the exogenous addition of ($1x10^2$ to $1x10^6$) eosinophils to preconstricted mesenteric arteries ($1x10^{-5}$M NE) from ΔdblGATA-1 mice was assessed in the presence and absence of PVAT. Eosinophils were purified from IL-5Tg spleens and assessed for purity by flow cytometry analysis as described. The response to eosinophil addition was measured as a percentage relaxation from the maximum constriction to baseline. In all experiments, eosinophils were added in a buffer containing $1x10^{-5}$M NE to avoid effects of NE-dilution on recipient arteries.

To establish whether eosinophils interacted directly or indirectly (via release of an eosinophil-derived factor), NE-stimulated eosinophils were centrifuged at 400g for 5 min and resulting supernatant passed through a 0.2µM filter before addition to NE-preconstricted WT or ΔdblGATA-1 vessels. Changes in vessel tension were compared with time controls, whereby bath solution was replaced by PSS+NE ($1x10^{-5}$M).

Addition of 10,000 purified eosinophils (described above) was used to delineate the mechanism involved in mediating eosinophil-derived relaxation of preconstricted mesenteric arteries from ΔdblGATA-1 mice. Adiponectin blocking peptide (5µg/mL) (Enzo Life Sciences)[5, 8, 11], L-NMMA ($1x10^{-5}$M) [6] (Sigma-Aldrich), and anti-IL-4 (0.4µg/mL) (Peprotech) were used to inhibit adiponectin, nitric oxide (NO) and IL-4 respectively. IgG isotype control (0.4µg/mL) (Peprotech) was used as a control for IL-4; D-NMMA (1x10-5 M, Sigma-Aldrich) as a control for L-NMMA and a control peptide was used for adiponectin (5µg/mL, Enzo Life Sciences). Intrinsic time controls were included for each inhibitor. To address the role of adrenoreceptors, we used propranolol (1µM), phentolamine (0.1µM, both Sigma-Aldrich), SR-592,30A (0.3µM, R&D Systems) CL-316, 243 (10µM, R&D Systems) and AMPT (3mM, Sigma-Aldrich). All inhibitors were incubated for 30 min at 37°C with either eosinophils only, arteries±PVAT, or both eosinophils and arteries (±PVAT). Both eosinophils and vessels were treated with NE ($1x10^{-5}$M) before addition of eosinophils to the vessels. The response to eosinophil addition was measured as a percentage relaxation.

Histological characterization of PVAT

Mesenteric adipose tissue was removed and fixed with adapted neutral buffered formalin (4% v/v formaldehyde (BDH), 0.154M NaCl (Sigma-Aldrich), 2% v/v glacial acetic acid (BDH), 1.37µM hexadecyl trimethyl-ammonium bromide (Sigma-Aldrich) for 24 hrs. Fixed tissues were dehydrated, cleared in xylol and infiltrated with paraffin in a dehydration automat (Citadel 2000, Shandon) using a standard protocol. Specimens were embedded in paraffin (Histocentre2, Shandon), sectioned on a

microtome (5µm sections). Prior to staining slides were deparaffinised with citroclear and rehydrated through alcohol (100% to 70%) to water. Haematoxylin & eosin (H&E) staining was performed by staining in Harris haematoxylin (Sigma-Aldrich), differentiation in acidified alcohol (1% HCl (Sigma-Aldrich) in 70% ethanol) and counterstaining with Eosin Y (Sigma-Aldrich) prior to dehydration and mounting using Depex mountant (BDH Laboratory Supplies).

Immunocytochemistry for Tyrosine Hydroxylase

Cytospins of splenic eosinophils were isolated from IL-5 Tg mice, purified by immunoisolation (>95% pure), and fixed in 4% paraformaldehyde. Immunocytochemical analysis was performed following permeabilisation (0.1% Triton-X in PBS, 10 minutes) using the rabbit polyclonal primary antibody to tyrosine hydroxylase (0.02mg/mL, 1h, room temperature) (AbCam), followed by a Texas Red-conjugated goat anti-rabbit IgG (H+L) secondary antibody (0.005mg/mL, 1h, room temperature) (Life Technologies), and mounted using DAPI-containing mounting medium (Vectorlabs). IgG controls were performed alongside. Images were captured using the Leica DM5000B microscope with DFC 3000G camera and Leica application suite-X software.

Study approval

Procedures were performed in accordance with the United Kingdom Animals (Scientific Procedures) Act of 1986, ethical permission from the Local Ethical Committee at Lund University, Sweden, and the University of Manchester Institutional Guidelines and conformed to the Directive 2010/63/EY of the European Parliament.

Supplemental References

1. Wang CY, Liao JK. A mouse model of diet-induced obesity and insulin resistance. *Methods Mol Biol*. 2012;821:421-433
2. Dyer KD, Moser JM, Czapiga M, Siegel SJ, Percopo CM, Rosenberg HF. Functionally competent eosinophils differentiated ex vivo in high purity from normal mouse bone marrow. *J Immunol*. 2008;181:4004-4009
3. Feng M, Whitesall S, Zhang Y, Beibel M, D'Alecy L, DiPetrillo K. Validation of volume-pressure recording tail-cuff blood pressure measurements. *Am J Hypertens*. 2008;21:1288-1291
4. Zhao X, Ho D, Gao S, Hong C, Vatner DE, Vatner SF. Arterial pressure monitoring in mice. *Curr Protoc Mouse Biol*. 2011;1:105-122
5. Greenstein AS, Khavandi K, Withers SB, Sonoyama K, Clancy O, Jeziorska M, Laing I, Yates AP, Pemberton PW, Malik RA, Heagerty AM. Local inflammation and hypoxia abolish the

protective anticontractile properties of perivascular fat in obese patients. *Circulation*. 2009;119:1661-1670

6. Withers SB, Simpson L, Fattah S, Werner ME, Heagerty AM. Cgmp-dependent protein kinase (pkg) mediates the anticontractile capacity of perivascular adipose tissue. *Cardiovasc Res*. 2014;101:130-137
7. Mulvany MJ, Halpern W. Contractile properties of small arterial resistance vessels in spontaneously hypertensive and normotensive rats. *Circ Res*. 1977;41:19-26
8. Dubrovska G, Verlohren S, Luft FC, Gollasch M. Mechanisms of adrf release from rat aortic adventitial adipose tissue. *American journal of physiology. Heart and circulatory physiology*. 2004;286:H1107-1113
9. Gao YJ, Lu C, Su LY, Sharma AM, Lee RM. Modulation of vascular function by perivascular adipose tissue: The role of endothelium and hydrogen peroxide. *Br J Pharmacol*. 2007;151:323-331
10. Lynch FM, Withers SB, Yao Z, Werner ME, Edwards G, Weston AH, Heagerty AM. Perivascular adipose tissue-derived adiponectin activates bk(ca) channels to induce anticontractile responses. *American journal of physiology. Heart and circulatory physiology*. 2013;304:H786-795
11. Oriowo MA. Perivascular adipose tissue, vascular reactivity and hypertension. *Med Princ Pract*. 2015;24 Suppl 1:29-37
12. Withers SB, Agabiti-Rosei C, Livingstone DM, Little MC, Aslam R, Malik RA, Heagerty AM. Macrophage activation is responsible for loss of anticontractile function in inflamed perivascular fat. *Arterioscler Thromb Vasc Biol*. 2011;31:908-913

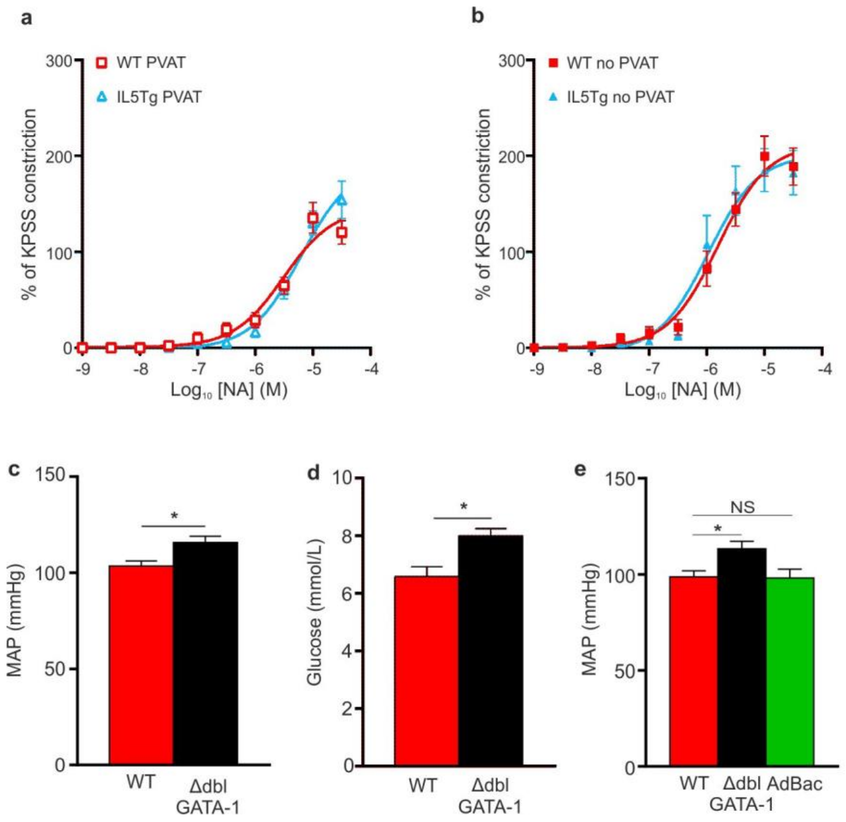

Figure 2 – figure supplement 1. Vascular reactivity of IL-5Tg mesenteric arteries and blood pressure and glucose tolerance in eosinophil deficient mice (a, b) NA-induced constriction of mesenteric arteries from IL-5Tg and WT mice **(a)** with or **(b)** without PVAT (IL-5Tg: n=11; WT: n=15; two-way ANOVA) as measured by wire myography **(c)** Peripheral MAP analysis by tail-cuff measurement of WT and ΔdblGATA-1 mice (WT: n=10, ΔdblGATA-1: n=5; *P=0.046, Mann Whitney test). **(d)** Blood glucose analysis of WT and ΔdblGATA-1 mice (mean±SEM; WT: n=6, ΔdblGATA-1: n=5: *P=0.0106, Student's t-test). **(e)** Peripheral MAP analysis of WT, ΔdblGATA-1 and AdBac mice (WT: n=5, ΔdblGATA-1: n=10 and AdBac mice: n=5; *P=0.025, one-way ANOVA).

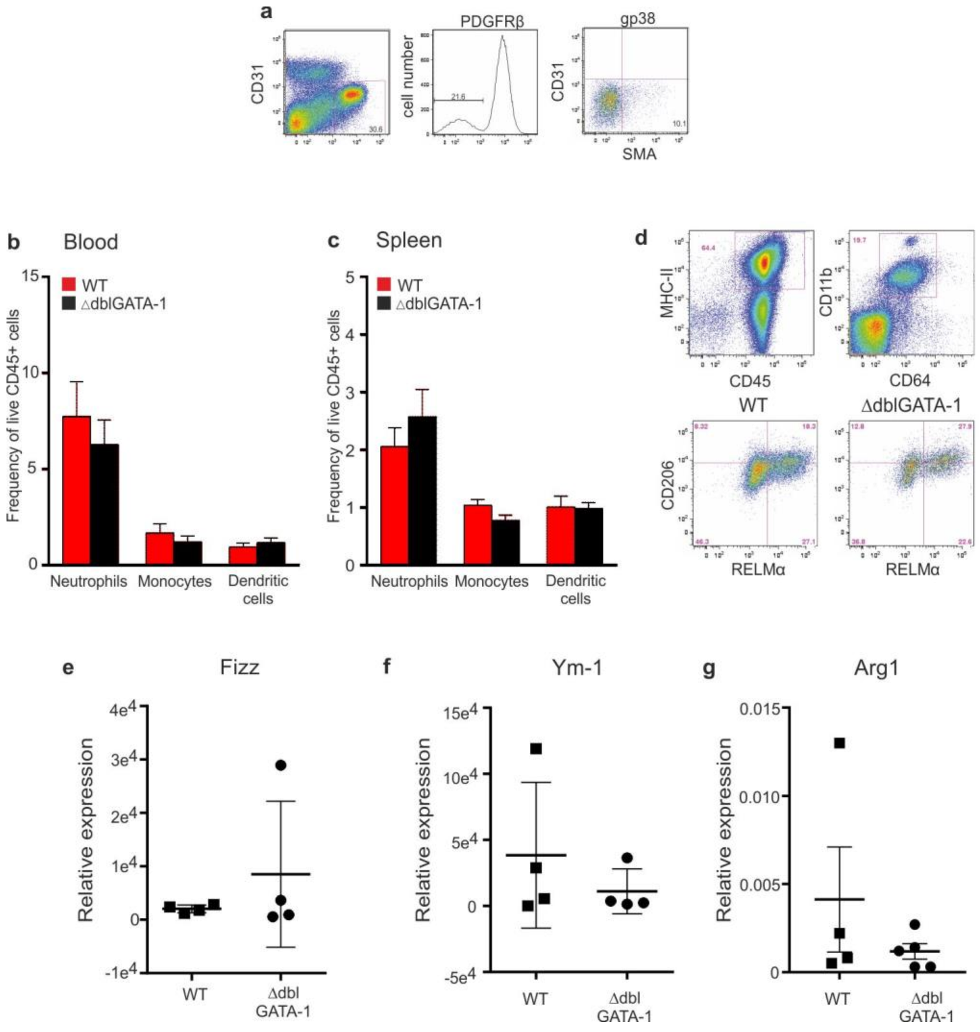

Figure 2 – figure supplement 2. Eosinophil mediated effects on PVAT are independent of pericytes and macrophages. (a) Flow cytometry gating strategy of SMA$^+$ pericytes (defined as live CD45$^-$ CD31$^-$ PDGFRβ$^+$ gp38$^-$ SMA$^+$ cells). Plots are pre-gated on live CD45$^-$ cells. (b) Flow cytometry analysis of the blood and (c) spleen of ΔdblGATA-1 and WT mice showed no significant differences in the proportions of dendritic cells, neutrophils and monocytes between strains (mean±SEM; n=4 and 7 for WT and ΔdblGATA-1, respectively). (d) Gating strategy for flow cytometry analysis of digested mesenteric adipose tissue to identify total and RELMα$^+$ CD206$^+$ macrophages in mesenteric adipose tissue of WT and ΔdblGATA-1 mice. qPCR analysis of (e) *Fizz-1* (f) *Ym-1* (WT: n=4, ΔdblGATA-1: n=4; P=NS, Student's t-test; one of two independent experiments), and (g) *Arg-1* in mesenteric adipose tissue (WT: n=4, ΔdblGATA-1: n=5; P=NS).

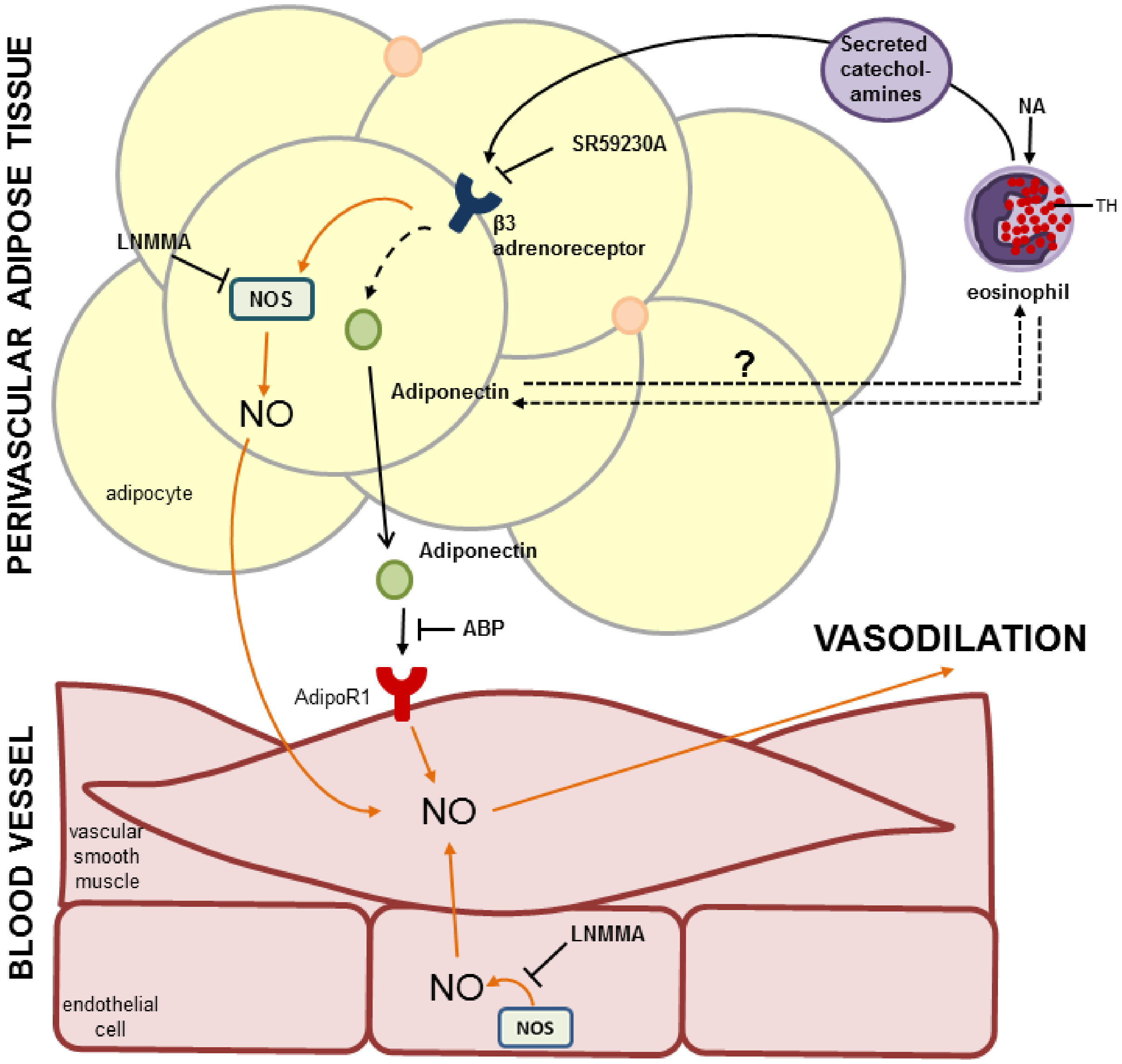

Figure 5 – figure supplement 1. Proposed schematic of eosinophil-mediated vascular relaxation. Eosinophils express tyrosine hydroxylase and produce and secrete catecholamines. Catecholamines promote β3 adrenoreceptor-induced activation of PVAT and can be blocked using the selective inhibitor SR-592,30A. Downstream vessel relaxation is mediated via adiponectin- and nitric oxide (NO)-dependent mechanisms, as relaxation is inhibited by the addition of ABP (adiponectin blocking peptide) and L-NMMA (a NO-signalling inhibitor). Adiponectin can be produced by PVAT and eosinophils, and may in turn act on eosinophils themselves. Whilst eosinophil derived NO does not appear to be critical, NO-production occurs via PVAT and blood vessel endothelial cells. Relaxation of the vessel is mediated at the PVAT (comprised of adipocytes and immune cells including eosinophils in control mice) and at the vessel level (comprising primarily of smooth muscle/endothelial cells).
